# *Heterobasidion* spp, triggers non-specific defence responses in bark of Norway spruce

**DOI:** 10.1186/1753-6561-5-S7-O30

**Published:** 2011-09-13

**Authors:** Malin Elfstrand, Jenny Arnerup, Karl Lundén, Marie Danielsson, Marten Lind, Ake Olson, Anna-Karin Borg-Karlsson, Jan Stenlid

**Affiliations:** 1 Dept of Forest Mycology and Pathology, Swedish University of Agricultural Sciences, Uppsala, Sweden; 2 Ecological Chemistry Group, Department of Chemistry, KTH, Stockholm, Sweden

## 

Norway spruce [*Picea abies* (L.) Karst.] is one of the economically most important conifer species in Europe. The major pathogen on Norway spruce is *Heterobasidion parviporum* (Fr.) Niemelä & Korhonen. The completed genome sequence of *H. irregulare* opens up new possibilities to understand the interactions between *Heterobasidion* spp. and spruce. However to date there are no completed genome sequencing projects of conifer genomes available for complementing studies. To achieve a better understanding of induced transcriptional defence responses in Norway spruce upon *Heterobasidion* spp. attack, we compared transcriptional responses in bark to *H. parviporum* infection to the response to wounding using cDNA-AFLP and transcriptome sequencing.

In an initial study bark samples were harvested at 3, 7 and 14 days post inoculation (dpi) and untreated bark was used as negative control. About 2500 transcribed derived fragments (TDFs) generated by cDNA-AFLP were screened. 199 TDFs were investigated further based on band intensity in the inoculated bark in relation to either untreated bark or wounded bark. Out of these, 119 TDFs had a putative homology and a consistent band intensity pattern between replications. A majority of these TDFs showed homology to genes known to associate with defence e.g. *3-deoxy-d-arabino-heptulosonate 7-phosphate synthetase* (*DAHP*), *Pathogenesis-related protein 1* (*PR1*), *Lipoxygenase* (*LOX*), *ACC-synthase* (*ACS*), *ACC-oxidase* (*ACO*) and *Jasmonate ZIM-domain 1* (*JAZ1*). Many of these are found in Salicylic acid- or Jasmonic acid/ethylene-signalling pathways. The majority of the TDFs showed a similar expression pattern for all treatments but samples inoculated with *H. parviporum* generally showed an enhanced reaction (induction/repression) compared to wounding alone. Expression patterns were confirmed by qPCR in material treated with wounding and inoculation with *H. parviporum* or *Phlebiopsis gigantea.* Our data suggest that infection with *H. parviporum* in Norway spruce induces a broad defence, with many similarities to non-specific defence responses in angiosperms. Additionally signs of reallocation of carbon from primary to secondary metabolism were evident*.*

With this information at hand we analysed four Norway spruce genotypes with either high or low susceptibility to *Heterobasidion* spp. [1] sampled 0, 5, 15 and 28 dpi with *H. annosum*. The bark phenol-composition was profiled in each sample. The 500,000 454-reads were assembled into 17,228 contigs that assembled in 14,364 putative transcript units (PTU) using the sequence assembler software Newbler**™** (http://www.454.com). The assembled reference file was annotated with the software Blast2Go and the PTUs were submitted to differential expression analysis. Data on associations between gene expression levels and phenol composition in bark upon *H. annosum* inoculation and level of susceptibility will be presented.

## References

[B1] Karlsson B Swedjemark G Genotypic variation in natural infection frequency of Heterobasidion spp. in a *Picea abies* clone trial in southern Sweden Scand J. Forest Research 2006 21 108 114 10.1080/02827580500529969

